# NooQ5 Supplementation Promotes Myogenic Differentiation in C2C12 Cells Under Chemical and Oxidative Stress Models

**DOI:** 10.3390/cells15191719

**Published:** 2026-09-22

**Authors:** Kirsty Ximena Noboa Carrasco, Cristhian David Andrade Alfaro, Ana Luísa Cremonese Lubiana, Roberta Viana Ferreira, Erika Cristina Jorge

**Affiliations:** 1 Departamento de Morfologia, Instituto de Ciências Biológicas, Universidade Federal de Minas Gerais, Av. Pres. Antônio Carlos, 6627, Belo Horizonte CEP 31270-901, MG, Brazilcd.andrade.alfaro@gmail.com (C.D.A.A.);; 2 AND Biotech AG, Marignanostrasse 85, 4059 Basel, Basel Stadt, Switzerland

**Keywords:** dietary nucleotides, myotube regeneration, oxidative stress, myogenic differentiation

## Abstract

Dietary nucleotides offer metabolic and antioxidant benefits, yet their direct impact on skeletal myogenesis and structural recovery remains to be fully elucidated. This study evaluated the comparative efficacy of the nucleotide formulation NooQ5 against creatine monohydrate in modulating differentiation, cellular stress, and regeneration in C2C12 myoblasts. Initial screening established a dose-dependent response, where 500 mg/L promoted cell proliferation, whereas 100 mg/L improved cell differentiation, without compromising cell viability. In uninjured cells, NooQ5 significantly accelerated early mTOR and S6 ribosomal protein phosphorylation within 1 h relative to creatine, driving downstream desmin accumulation by 72 h. Under H_2_O_2_-induced oxidative stress, NooQ5 preserved membrane integrity by reducing lactate dehydrogenase (LDH) release compared to creatine and modulating intracellular glutathione redox balance. Furthermore, following chemical injury with BaCl_2_, as a muscle injury model, NooQ5 maintained a high fusion index, promoting myotube hypertrophy. Overall, these findings demonstrate that the nucleotide ratio in NooQ5 enhances early protein synthesis kinetics and protects cell membrane integrity, showing potential as an alternative for muscle bioenergetics and structural remodeling in C2C12 myotubes.

## 1. Introduction

Beyond their well-established role as the structural backbones of DNA and RNA, nucleotides independently exert critical functions in modulating cell growth and regulating cellular energy expenses. Structurally, these molecules consist of a pentose sugar (ribose or deoxyribose), a nitrogenous base (either a purine or a pyrimidine), and a phosphate group [1,2]. Rather than functioning solely as organized frames for genomic information, individual nucleotides act as autonomous metabolic hubs. For instance, adenosine triphosphate (ATP) serves as the primary cellular energy currency, while cyclic adenosine monophosphate (cAMP) and cyclic guanosine monophosphate (cGMP) operate as essential secondary messengers and enzymatic cofactors [2]. During cell proliferation, the rapid expansion of biomass demands an immense influx of raw building blocks; since nucleotides form the structural foundation of this process, their availability is tightly governed by complex, multilevel metabolic regulations. Furthermore, the in vivo synthesis of purines and pyrimidines represents a highly energy-intensive process driven by ATP consumption [3]. Reliance on exogenous nucleotides mitigates cellular reliance on de novo synthesis pathways [4].

Beyond the physiological roles of endogenous nucleotides, dietary nucleotides have been widely documented for their multifaceted biomedical functions, including antioxidant properties, metabolic modulation, immune response enhancement, and cellular signaling regulation [1,5,6,7]. Moreover, exogenous nucleotide availability spares critical amino acids like glutamine, aspartate, and glycine from being channeled into nucleotide synthesis, thereby redirecting them toward primary physiological growth pathways [5].

Biomedical applications of commercial nucleotide formulations have been documented at daily dosages ranging from 50 mg to approximately 500 mg across populations spanning from competitive athletes to older adults [8,9,10,11]. In these clinical settings, the primary physiological outcomes observed include enhanced physical recovery, attenuated serum creatine kinase (CK) levels, and promotion of healthy aging trajectories. Furthermore, translational research in animal models has demonstrated that exogenous nucleotide administration effectively suppresses oxidative stress and modulates gut microbiota composition [12,13,14,15].

To date, investigations evaluating the effects of dietary nucleotides in skeletal muscle have been largely restricted to livestock production to enhance meat quality, demonstrating improved outcomes such as reduced adipose tissue deposition and enhanced growth, primarily driven by optimized energy utilization and protein synthesis [16,17,18,19]. At the cellular level, research has focused on the individual administration of cytidine monophosphate (CMP) and uridine monophosphate (UMP) to mitigate C2C12 myotube atrophy in models of sarcopenia [20]. However, the multi-nucleotide formulation evaluated in the present study, herein designated NooQ5, features a trade secret blend ratio of AMP, GMP, CMP, UMP, and inosine monophosphate (IMP), specifically designed to be implemented as an ergogenic supplement for athletic performance. Consequently, evaluating this formulation against creatine, the established biomedical gold standard for muscle bioenergetics and protein synthesis [21,22,23], will determine its comparative efficacy in accelerating cellular recovery and modulating muscle mass dynamics.

Given that the metabolic advantages of exogenous nucleotides are more pronounced during intense structural remodeling, experimental models of muscle injury and regeneration provide an ideal framework to test this formulation. To simulate these conditions in vitro, the previous literature has established the use of hydrogen peroxide (H_2_O_2_) at concentrations ranging from 100 µM to 4 mM to induce targeted cellular oxidative stress [20,24,25]. Within the pathophysiology of muscle injury, excessive oxidative stress acts as a primary driver of cellular damage, disrupting membrane integrity and severely impairing the bioenergetic machinery required for repair [26]. To comprehensively evaluate how the NooQ5 formulation modulates these interconnected processes of damage, inflammation, and recovery, this study utilized a barium chloride (BaCl_2_) injury model in cultured myotubes. BaCl_2_ induces muscle damage via calcium-activated proteolysis and has gained widespread attention due to its high reproducibility in altering the muscle phenotype [27], making it a robust and reliable tool for in vitro muscle injury and regeneration dynamics [28,29,30].

Given these considerations, exogenous nucleotide supplementation can optimize cellular energy production and protein synthesis during the demanding phases of muscle differentiation and repair. Therefore, the aim of this study is to elucidate the effects of NooQ5 supplementation in in vitro models of muscle hypertrophy and hyperplasia, establishing a mechanistic foundation prior to its application in vivo models. Furthermore, this study evaluates whether this proposed formulation can potentially outperform traditional energetic supplements such as creatine.

## 2. Materials and Methods

### 2.1. Cell Culture Conditions

Murine C2C12 myoblasts were maintained in growth medium (GM) consisting of Dulbecco’s Modified Eagle Medium (DMEM) High Glucose (Gibco, Cat #12100046, Waltham, MA, USA), supplemented with 10% fetal bovine serum (FBS, Gibco, Cat #12657-029, Brazil qualified) and 1% antibiotic-antimycotic solution (Gibco). For specific experimental conditions, starvation medium (SM) contained DMEM High Glucose, 0.5% FBS, and 1% anti-anti, and differentiation medium (DM) contained DMEM High Glucose, 2% horse serum (HS, Gibco), and 1% anti-anti. Culture media containing different additives were prepared in DMEM and sterilized by filtration through a 0.22 μm membrane filter.

For all experimental assays, cells were seeded at a density of 2 × 10^4^ cells/cm^2^ in GM. Supplementation and subsequent testing were initiated when the cell culture reached approximately 70% confluence at 24 h.

### 2.2. Supplement Dosage Establishment

The experimental treatment, NooQ5, was supplied by AND Biotech AG. NooQ5 is a proprietary ingredient of the commercial supplement NooQ™ Muscle Charge (AND Biotech AG, Basel, Switzerland). However, all experiments in this study were conducted using the isolated nucleotide fraction alone. The optimal concentration of the NooQ5 supplement was determined by evaluating a range of concentrations at 5, 1, 0.5, 0.1, and 0.05 g/L. Various media conditions of NooQ5 were screened in both GM and SM. Cell viability was assessed using the MTT assay (Gibco) following a 2 h incubation period, after which the resulting formazan crystals were solubilized with acidified isopropanol. Absorbance at 595 nm was measured using an ELx800 microplate reader (BioTek Instruments, Winooski, VT, USA), and the results were expressed as the cell viability percentage relative to the control group.

Based on these initial screening results, the concentrations demonstrating the most favorable effects were selected for a 5-day cell viability evaluation in both GM and DM.

### 2.3. Morphological Analysis via Immunocytochemistry (ICC)

To evaluate the effects of the supplement on myogenic differentiation, ICC was performed following treatment with NooQ5 at concentrations of 100 and 500 mg/L in DM. Cells were fixed with 4% paraformaldehyde (PFA; Sigma-Aldrich, Cat# P6148, Burlington, VT, USA) for 15 min at room temperature and washed three times with PBS. Endogenous peroxidase activity was quenched by incubation with 1% (*v*/*v*) H_2_O_2_ for 5 min. After this, samples were permeabilized with 0.2% PBS-Tween 20 (PBST). Subsequently, samples were blocked with 5% bovine serum albumin (BSA) for 1 h. Cells were then incubated overnight at 4 °C with Myosin heavy chain (MF20; 1:100; Developmental Studies Hybridoma Bank—DSHB, IA, USA). After three sequential washes with PBS for 5 min each, samples were incubated with the Dual Link System anti-mouse/anti-rabbit secondary antibody (EnVision+ Dual Link, Dako, Cat# K4061, Glostrup, Denmark) for 90 min at room temperature. After another series of washes, antigen visualization was performed using a 3,3′-diaminobenzidine (DAB; Biotium, Cat# 30015, Fremont, CA, USA) substrate kit. Nuclei were counterstained with hematoxylin for 1 min, followed by thorough rinsing with distilled water. To preserve the staining, a final post-fixation step with 4% PFA was performed. Images were acquired using an inverted bright-field light microscope (AE31, Motic, Xiamen, China) equipped with a 10× objective (Numerical Aperture (NA) = 0.25).

### 2.4. Additives Treatment Design

To evaluate the cellular effects of the selected NooQ5 concentrations, cells were analyzed via the MTT assay. For comparative analysis, a parallel group was treated with micronized creatine monohydrate (Lot 04320426, Healthy Labs, Brazil) at a concentration of 600 mg/L as a reference control. Cells kept in the respective basal media without any additives served as the negative vehicle control.

### 2.5. Immunoblotting

To evaluate the temporal effects of the supplements on protein expression, cells were treated in DM for 1, 24, and 72 h. Total protein was extracted by harvesting the cells in an ice-cold RIPA buffer supplemented with phosphatase cocktail (Sigma-Aldrich, Cat. #S6508; Sigma-Aldrich, Cat. #221368), protease inhibitor cocktails (Sigma-Aldrich, Cat. #S8820), and 1% (*v*/*v*) dithiothreitol (DTT; Roche, Cat. #10197777001, Basel, Switzerland), using a cell scraper on ice. The cell lysates were then subjected to mechanical disruption using a bead-beater (L-Beader 6, Loccus, São Paulo, Brazil) for three cycles at 3500 rpm for 30 s, with 1 min intervals on ice between cycles. The homogenates were centrifuged at 12,000× *g* for 10 min at 4 °C, and the supernatants were collected. Protein concentration was quantified using the Bradford assay (Bio-Rad, Cat. #5000006, Shinagawa City, Japan); absorbance at 595 nm was measured using an ELx800 microplate reader (BioTek Instruments).

For immunoblot analysis, 20 µg of total protein per lane was resolved using 6% and 14% SDS-PAGE gels (Sodium Dodecyl Sulfate–Polyacrylamide Gel Electrophoresis) with *N*,*N*′-methylene-bis-acrilamide (Sigma-Aldrich, Cat. M7279). The samples were diluted with Laemmli buffer at a 1:1 ratio. Electrophoresis was performed at 70 V through the stacking gel and increased to 100 V for the resolving gel. Proteins were then transferred to membranes at 110 V for 1.5 h or 2.5 h at 90 V for 14% and 6% gels, respectively. Transfer effectiveness was visually confirmed via Ponceau S 0.1% staining. Membranes were blocked with BSA 5% diluted in Tris-Buffered Saline with Tween-20 (TBST) and subsequently incubated overnight at 4 °C with primary antibodies against mTOR (1:500; Cell Signaling Technology, Cat. #2983, Danvers, MA, USA), Phospho-mTOR (Ser2448) (1:500; Cell Signaling Technology, Cat. #2971), S6 Ribosomal Protein (1:1000; Cell Signaling Technology, Cat. #2217S), Phospho-S6 Ribosomal Protein (Ser240/244) (1:1000; Cell Signaling Technology, Cat. #2215), MyoD (1:500; Invitrogen, Cat. #MA5-12902, Waltham, MA, USA) and desmin (1:1000; Cell Signaling Technology, Cat. #5332). Following primary incubation, membranes were washed three times with TBST and probed with the anti-mouse/anti-rabbit secondary antibody (Dako, Cat# K4061, Glostrup, Denmark) for 1.5 h. Protein bands were visualized using the DAB detection system. Quantitative analyses were performed via Fiji.

### 2.6. Establishment of Experimental Stress Models

To determine the optimal scheme and concentrations for oxidative stress via H_2_O_2_ (Merck, Cat# 88597, Saint Louis, MO, USA) and BaCl_2_ (Sigma-Aldrich) for the myotube damage model, fully differentiated myotubes were utilized.

For the oxidative stress model, a reduction in cell viability of up to 20% was established as the critical threshold limit. Myotubes were exposed to DM supplemented with H_2_O_2_ at concentrations of 50, 75, 100, and 500 µM. Based on these preliminary results, a concentration of 100 µM H_2_O_2_ was selected for subsequent evaluation in combination with the additives.

The injury and supplementation scheme was determined via morphological evaluation of the cell cultures. Differentiated cells were treated with BaCl_2_ at concentrations of 0.48% and 0.6% (%*w*/*v*) prepared in DM. Following a 6 h incubation period, the damage medium was replaced with either DM or GM. In the case of GM-treated groups, the medium was replaced with DM after 2 days to re-induce myogenic differentiation.

### 2.7. Evaluation of Oxidative Stress Mitigation

To evaluate the protective effects of the supplements against oxidative stress, differentiated myotubes cultured in DM served as the untreated control group, while *N*-acetyl-L-cysteine (NAC; Sigma-Aldrich, Cat. #A9165) was utilized as a positive antioxidant control alongside the NooQ5 and creatine treatment groups.

The intracellular glutathione disulfide to reduced glutathione (GSSG/GSH) metabolic ratio was quantified 4 h post-oxidative challenge using a commercial kit (Sigma-Aldrich, Cat. #MAK440), following the manufacturer’s instructions. To assess cell membrane integrity and the recovery potential of the additives, lactate dehydrogenase (LDH) activity was measured in the culture supernatant 6 h after H_2_O_2_ exposure using an LDH assay kit (Sigma-Aldrich, Cat. #MAK066) according to the manufacturer’s protocol. Additionally, cell viability was evaluated via the MTT assay 24 h post-treatment to determine the protective capacity of the supplements against 100 µM H_2_O_2_-induced cytotoxicity.

### 2.8. Evaluation of Myotube Damage and Regeneration

To evaluate the protective and regenerative effects of the supplements against BaCl_2_-induced damage, differentiated myotubes cultured in basal DM served as the untreated control group, while Insulin-like Growth Factor 1 (IGF-1; Sigma-Aldrich, Cat. # I3769) was utilized as a positive reference control alongside the NooQ5 and creatine treatment groups.

### 2.9. LDH Activity and Creatine Kinase (CK) Assays

To assess membrane leakage and acute cytotoxicity, cell culture supernatants were collected at 6, 24, 48, and 96 h post-injury. LDH activity was quantified. For long-term recovery assessment, supernatants were collected at 24, 48, 96, and 144 h post-injury and stored at −80 °C until analysis. CK activity was subsequently determined via ELISA using the mouse muscle creatine kinase kit (ELK Biotechnology, Cat. #ELK2301, Wuhan, China) following the manufacturer’s instructions.

### 2.10. Gene Expression Analysis

Due to severe cellular stress following BaCl_2_ exposure, total protein yield was insufficient for immunoblot analysis at 1 h; therefore, cellular recovery was evaluated at the transcriptional level via a quantitative polymerase chain reaction (qPCR). Total RNA was isolated by harvesting cells in TRI-reagent (Sigma-Aldrich) following the manufacturer’s instructions. cDNA synthesis was performed using a commercial reverse transcription kit, High-Capacity cDNA Reverse Transcription Kit (Thermo Fisher Scientific, Cat. # 4368814, Vilnius, Lithuania). qPCR was executed on a Rotor-Gene Q real-time PCR cycler (Corbett Research, Venlo, The Netherlands) with QuantiNova^®^ SYBR^®^ Green PCR Kit (Qiagen, Hilden, Germany). Relative mRNA expression levels of *MyoD* (GTGGCAGCGAGCACTACA and GACACAGCCGCACTCTTC) and *desmin* (GTGGAGCGTGACAACCTGAT and ATGTTCTTAGCCGCGATG GT) were calculated using the comparative ΔΔCt method, with glyceraldehyde 3-phosphate dehydrogenase (*Gapdh*; AGGTCGGTGTGAAGGGATTT and TGTAGACCATGTAGTTGAGG) utilized as the internal housekeeping gene reference.

### 2.11. Immunofluorescence and Fusion Index Quantification

Cells were seeded on coverslips (0.13–0.16 mm) and incubated for 48, 96 and 144 h. After discarding the media, coverslips were washed with PBS and fixed with 4% PFA for 10 min at room temperature and washed again with PBS. To quench background fluorescence, cells were incubated with glycine 1 mM for 10 min. Blocking and permeabilization were performed simultaneously using a solution containing 5% goat serum (Gibco), 0.1% Triton X-100 (Sigma), and 1% bovine serum albumin (BSA) in PBS for 1 h at room temperature. Membranes were then incubated overnight at 4 °C with MF20 (1:100; DSHB), followed by three washes with PBS containing 0.1% Tween-20 (PBS-T) and incubated with an Alexa Fluor 488 anti-mouse IgG secondary antibody (Invitrogen, Cat. # A21202) for 1 h 30 min at room temperature. After three additional PBS-T washes, nuclei were counterstained with DAPI (1:1000, Invitrogen). Coverslips were mounted using Fluoromount-G mounting medium (Invitrogen) and visualized under an Axio Vert.A1 microscope (Zeiss) equipped with a 40× objective lens (NA = 0.55). Micrographs were processed using ZEN software (Zeiss 3.12), and the fusion index was automatically quantified using the Cellpose (3.1+) deep-learning segmentation framework.

### 2.12. Statistical Analysis

Data are expressed as mean ± standard deviation (SD). Statistical analyses were performed using GraphPad Prism software (version 10.2.1). Data normality was assessed using the Shapiro–Wilk test. Comparisons between multiple groups were analyzed using one-way or two-way analysis of variance (ANOVA), followed by Tukey’s or Sidak’s multiple comparisons tests for parametric data, respectively. For non-parametric data, the Kruskal–Wallis test followed by Dunn’s multiple comparisons test was used. A *p*-value < 0.05 was considered statistically significant.

## 3. Results

### 3.1. Supplement Effect and Concentration Standardization

To evaluate the safe concentration range and potential cytotoxicity of NooQ5 supplementation, as well as its effects on C2C12 cell cultures, various concentrations (0.05 to 5 g/L) were tested in both GM and low-serum medium formulations (Figure 1B,C). A significant increase in cell viability was observed at higher concentrations (0.5 to 5 g/L) in GM, with the most pronounced effect occurring between days 1 and 3 at 0.5 g/L (Figure 1B). In contrast, culturing cells in SM led to a sharp reduction in cell viability at concentrations ranging from 0.5 to 5 g/L (Figure 1D). Notably, the 0.1 g/L concentration showed no statistically significant cytotoxicity, and more differentiated myotubes were morphologically visible under this condition. Based on these initial observations, the 0.5 and 0.1 g/L concentrations were selected for extended evaluation up to 5 days in both GM and DM (Figure 1C,E). These long-term analyses reinforce our previous findings. In the presence of serum (GM), NooQ5 at 0.5 g/L exerts a potential proliferative effect, whereas no significant differences in cell viability were observed in low-serum formulations (DM) on day 5. However, a distinct phenotypic pattern emerged regarding myotube formation. Immunocytochemistry analysis on day 3 revealed a higher density of myotubes in cells treated with 0.1 g/L compared to both the 0.5 g/L group and the untreated control (Figure 1F–H). Taken together, these results establish that while a concentration of 500 mg/L acts as an additive that promotes cell viability, a lower concentration of 100 mg/L successfully enhances myogenic differentiation without compromising cell viability. Consequently, as the primary focus of this study centers on myogenesis, the 100 mg/L (0.1 g/L) concentration was selected for all subsequent analyses due to its superior capacity to enhance differentiation. Nevertheless, the potential proliferative effect observed at 500 mg/L remains a compelling avenue for further investigation in future studies.

### 3.2. Comparative Impact of NooQ5 and Creatine

Given that creatine is a well-established and efficient supplement widely recognized for its capacity to induce myogenesis, we next investigated whether any comparative differences existed between our designated concentration of NooQ5 (100 mg/L) and creatine. When evaluating cell viability, no statistically significant differences were observed among the creatine, NooQ5, and untreated control groups in either GM or DM conditions (Figure 2A,B, respectively). In agreement with these findings, phase-contrast microscopy examination at day 3 of analysis revealed no apparent morphological alterations or variations in cell density among the experimental groups in GM (Figure 2E–G). A time-dependent increase in total protein abundance was evident in all treatment groups from 1 h to 72 h (Figure 2C,D).

### 3.3. Temporal Dynamics of NooQ5 and Creatine Supplementation

Following the concentration standardization and having verified no apparent differences during the initial screening of the additives, we analyzed the temporal dynamics to distinguish the specific effects of each supplement on early cellular mechanisms. At 1 h post-treatment medium change, no visual differences in early cell morphology were observed among the groups (Figure 3A–C). However, immunoblotting analysis of harvested proteins revealed early shifts in the mechanistic pathways involved in protein maturation. Specifically, signaling pathways related to protein synthesis, such as mTOR and S6, were modulated. The phosphorylation levels of both proteins were elevated in the NooQ5-supplemented medium (Figure 3D–O), with the increase in p-S6 being statistically significant, whereas creatine-treated cells showed lower levels. This trend was further confirmed by the relative phosphorylation ratios (phosphorylated/total protein), which were significantly higher in the NooQ5 group (Figure 3I,O). Consequently, to ensure accurate quantification, protein expression was normalized against total protein lane density visualized by Ponceau S staining (Figure 3F,H,L,N) rather than a single housekeeping reference protein.

Upon maintaining the cells in supplemented DM for 24 h, no apparent differences were observed in cell morphology (Figure 4A–C). Regarding MyoD appearance, no significant variations were found between the supplemented groups and the untreated control (Figure 4G–I). By 72 h, distinct phenotypic changes emerged in the cultures (Figure 4D–F), with both supplement groups displaying larger and more developed myotubes compared to the control. Notably, a higher presence of branched myotubes was characteristically observed in the NooQ5 group (Figure 4E). To evaluate whether this morphological enhancement correlated with structural protein synthesis, desmin expression was analyzed. While both NooQ5 and creatine treatments drove an overall increase in desmin levels by 72 h (Figure 4J–L), the accumulation was more pronounced in the additive’s cohort. This suggests that protein accumulation driven by accelerated synthesis becomes prominent in supplemented conditions by this time point. Regarding mTOR levels at 24 h, an increase was observed in the creatine group, presumably indicating a delayed activation of this pathway by this supplement. At 72 h, mTOR levels returned to baseline for both creatine and NooQ5.

### 3.4. Protective Effects of NooQ5 Against Oxidative Stress in C2C12 Myotubes

An oxidative injury model using H_2_O_2_ was first established to assess the protective capacity of the supplements during C2C12 differentiation. The screening procedures and timeline for defining the H_2_O_2_ injury model, along with its downstream analyses, were executed in accordance with the integrated experimental design shown in Figure 5A. A preliminary dose–response screening (50 to 500 µM) demonstrated a concentration-dependent decrease in cell viability (Figure 5B), which correlated with progressive morphological disruption and reduced myotube density (Figure 5C–F). Based on these screening results, a 100 µM concentration yielding approximately 20% cell mortality was selected for subsequent rescue experiments. This specific concentration was chosen to ensure a robust yet non-lethal injury model, as a 500 µM challenge would induce overwhelming cellular damage that could mask any potential protective effects during the short-term evaluation.

Under the oxidative stress environment, relative cell viability was significantly compromised across all H_2_O_2_-treated groups compared to the untreated control (Figure 5G). However, to assess early cellular protection and membrane integrity, LDH release and intracellular redox state were analyzed at 6 and 4 h post-treatment, respectively. Notably, NooQ5 supplementation demonstrated a profound protective effect on cell membrane integrity, yielding a significantly lower LDH activity (~60 U/L) compared to the creatine group (~90 U/L) (Figure 5I). While LDH release in the H_2_O_2_-only group did not reach statistical significance compared to treatments, baseline values remained low relative to both the positive control and NooQ5 groups compared to creatine. Regarding the intracellular antioxidant response at 4 h, the glutathione ratio exhibited distinct regulatory patterns among the treatments, with the NooQ5 group maintaining a lower ratio compared to both the creatine and positive control cohorts. By contrast, the H_2_O_2_-only control exhibited the most pronounced drop in GSH/GSSG, reflecting a clear impact of oxidative stress on this metabolic parameter (Figure 5H). Together, these results demonstrate that NooQ5 offers protection against H_2_O_2_-induced cell membrane damage during early differentiation phases.

### 3.5. NooQ5 Supplementation Promotes Myotube Rescue and Structural Preservation Post BaCl_2_-Exposure

To evaluate the capacity of the supplements to promote myotube recovery and rescue structural integrity following severe tissue damage, a BaCl_2_-injury model was utilized in accordance with the experimental timeline (Figure 6A). Following chemical injury, cells were transitioned to a supplemented GM for the first 48 h to stimulate early recovery dynamics with supplementation with NooQ5 and creatine, before shifting to a supplemented DM up to 144 h. Morphological tracking via immunofluorescence and structural quantification revealed distinct recovery patterns among the groups. While the BaCl_2_-only group exhibited a progressive, significant decline in the fusion index over time, NooQ5 supplementation successfully counteracted this degradation, maintaining a remarkably stable and high fusion index across 48, 96, and 144 h (Figure 6B). Furthermore, the total myotube relative area analysis highlighted a substantial differentiation advantage for the NooQ5 cohort (Figure 6C). At 144 h, NooQ5-treated cultures reached a significantly higher myotube area compared to both the BaCl_2_ control and the creatine group, which was visually corroborated by the presence of large, well-structured, multinucleated green-stained myotubes (Figure 6D–O). Characteristically, myotube formation in the IGF-1-positive control group remained constant without significant temporal variations, indicating that both NooQ5 and creatine actively drive myotube development over time.

To dissect the underlying molecular and biochemical mechanisms, gene expression patterns and enzymatic release were monitored at key points of the recovery phase. *MyoD* and *desmin* expression levels showed temporal modulations, with NooQ5 driving recovery peaks at 96 h post-injury (Figure 6Q). On the other hand, *MyoD* expression remained stable throughout the analysis. This trend aligns with our previous immunoblotting observations under non-injured conditions, where *MyoD* showed no significant variations (Figure 3D–F) at 24 h while *desmin* (Figure 3M–O) was already significantly upregulated at 72 h.

Biochemically, CK concentration peaked significantly at 96 h in the creatine group (Figure 6R). This was in concordance with cell membrane damage and cytotoxicity kinetics monitored via LDH activity, revealing that while the creatine group suffered a substantial spike in LDH release at 96 and 144 h, NooQ5 effectively controlled membrane leakage, keeping LDH activity significantly lower and stable throughout the entire long-term differentiation window (Figure 6S). Together, these findings demonstrate that NooQ5 performs comparably to creatine in preserving cell membrane integrity and accelerating morphological rescue following a BaCl_2_ injury in vitro.

## 4. Discussion

The first phase of this study established that the biological response to NooQ5 is highly dependent on the concentration administered during specific phases of cell culture. As indicated by the MTT assay, our results demonstrate that a concentration of 500 mg/L of NooQ5 maximizes mitochondrial metabolic activity in GM (Figure 1B,C), whereas a lower dose of 100 mg/L optimizes the differentiation profile in DM (Figure 1D,E). Interestingly, while 100 mg/L did not induce a significant increase in baseline cell viability in GM, it promoted myotube differentiation in DM without triggering cytotoxicity. This concentration-dependent threshold suggests that while rapidly dividing myoblasts benefit from a high nucleotide abundance to support DNA replication via the salvage pathway, differentiating myotubes are highly sensitive to nucleotide saturation.

This model is strongly supported by recent transcriptomic and proteomic data from human muscle biopsies collected after resistance exercise, which revealed that the most significantly upregulated metabolic pathways are those involved in DNA and RNA nucleotide biosynthesis [31]. In this case, resistance training increases key enzymes in purine synthesis, such as ATIC, NME2, GUK1 and PHGDH [32]. Biochemically, PHGDH initiates the serine synthesis pathway starting from glycolysis, in which serine is subsequently converted into glycine, a process that donates a carbon unit to the folate pool to generate 5,10-CH2-THF; it is a foundational precursor for the synthesis of adenine (A), guanine (G), and thymine (T) [33]. Conversely, for the pyrimidine nucleotides uracil (U) and cytosine (C), their de novo synthesis is dependent on this pathway. The serine/glycine pathway acts as a critical driver of mitochondrial bioenergetics and ATP production, which directly fuels the high-energy demands required for pyrimidine ring assembly [34]. In addition to de novo synthesis, cells can acquire purines and pyrimidines through salvage recycling pathways. G and A can be obtained by coupling them to phosphoribosyl pyrophosphate (PRPP) via the actions of hypoxanthine-guanine phosphoribosyltransferase (HGPRT) and adenine phosphoribosyltransferase (APRT), while pyrimidines are limited to the availability of free bases [35]. Given the bioenergetic cost of de novo nitrogenous base synthesis, supplementation with NooQ5 contributes significantly to cellular energy conservation by directly providing pre-assembled building blocks readily usable across multiple biosynthetic pathways. Nevertheless, it is critical to highlight that nucleotide oversaturation can trigger a severe homeostatic imbalance. Excessive nucleotide pools disrupt cellular proliferation through mechanisms linked to replication stress, which activates the ATR-dependent checkpoint pathway to arrest the cell cycle [4,36]. Consequently, the critical threshold concentration for proliferation was established at 500 mg/L in contrast to the 5 g/L dosage. Furthermore, as the differentiation phase apparently requires a lower nucleotide investment, the optimal concentration to support ongoing myogenesis was determined to be 100 mg/L in this in vitro model.

Currently, creatine is considered the gold standard supplement for muscle mass gains, which prompted our decision to compare its cellular effects against nucleotide supplementation. For this purpose, a concentration of 600 mg/L (4.5 mM) was selected for commercial creatine monohydrate, as previous studies have demonstrated its efficacy in inducing myotube formation in C2C12 cells at dosages up to 5 mM [37]. In line with the phenotypic observations, creatine supplementation showed no significant differences in cellular metabolic viability compared to NooQ5 under both GM and DM conditions (Figure 2). Despite this equivalent viability profile, crucial early divergent responses were detected in the phosphorylation kinetics of the mTOR/S6 axis just one hour post-media change. While mTOR phosphorylation levels in the creatine group showed no significant variation relative to the control, NooQ5 treatment induced a significantly higher activation. In the same manner, downstream ribosomal protein S6 phosphorylation was higher in the NooQ5 group, compared to both the control and creatine groups; notably, creatine-treated cells exhibited S6 phosphorylation levels that were even lower than the non-supplemented control (Figure 3D–O). These results suggest that while the mTOR pathway was in the initial stages of activation in the creatine group, as evidenced by its lack of variance relative to the control, this signaling had not yet propagated downstream to phosphorylation of S6. This rapid, early-phase activation by NooQ5 is likely driven by the direct signaling capacity of exogenous UMP and CMP present in the formulation. Emerging evidence demonstrates that the availability of pyrimidine nucleotides, particularly uridine, exerts a synergistic, direct stimulatory effect on mTORC1 signaling that operates independently of general amino acid availability [37,38,39]. In opposition, creatine uptake relies heavily on specific transporter dynamics (CreaT), primarily serving to refill intracellular phosphocreatine pools and stabilize bioenergetics upon entry [38,40]. These findings indicate that the immediate availability of pre-assembled nucleotide building blocks triggers a more efficient and timely activation of upstream myogenic pathways, consistent with previous reports [41,42].

As differentiation progressed toward 24 h and 72 h, the expression dynamics of myogenic regulatory markers provided deeper insights into myotube maturation. At 24 h, MyoD appearance remained uniform across all experimental groups (Figure 4A–C,G–I), indicating a synchronized entry into the myogenic commitment phase. However, by 72 h, a distinct phenotype began to emerge. While desmin levels did not reach statistical significance, a clear upward trend was observed in both the NooQ5 and creatine-supplemented groups compared to the non-supplemented control (Figure 4D–F,J–L). This biochemical trend was strongly corroborated by morphological examination, which revealed qualitatively more pronounced, aligned, and well-defined myotubes in the supplemented cultures (Figure 4E,F). Desmin is a muscle-specific intermediate filament essential for maintaining structural integrity and anchoring myofibrils during advanced differentiation [43]. Therefore, the cooperative rise in desmin expression alongside the enhanced visual presence of myotubes suggests that both NooQ5 and creatine act as accelerating agents for structural remodeling and cytoskeletal consolidation during the late stages of myogenesis.

mTOR activation has been linked to reduced longevity [44,45]. As a result, inhibiting mTOR using rapamycin has become a major focus for preventing age-related diseases [46,47]. While persistent overactivation of mTOR is considered disadvantageous, in the present study, mTOR activation at 24 h was significantly lower in the NooQ5 group compared to both the control and creatine groups. Furthermore, by 72 h post-treatment, pathway activity for both additives returned to baseline relative to the control. These findings suggest an earlier, transient activation of the mTOR pathway by NooQ5 relative to creatine, followed by deactivation at 72 h, indicating no sustained effects that would adversely impact longevity.

Considering that no statistically significant phenotypic differences were observed under baseline conditions, it is important to note that the primary physiological benefits of nucleotide and creatine supplementation are typically manifested under conditions of metabolic demand or physical stress, such as resistance training [31]. Consequently, we hypothesized that cellular injury models such as H_2_O_2_ and BaCl_2_ could be implemented to study the influence of supplementation in myotubes under structural damage.

To evaluate cellular resilience under oxidative stress, we first established an injury threshold using 100 µM of H_2_O_2_, which induced a ~20% reduction in cell viability (Figure 5B–F). Remarkably, when exposed to this oxidative stimulus for 24 h, NooQ5 supplementation effectively mitigated cell death, restricting viability loss to approximately 23%, compared to 27% in the H_2_O_2_ group and 33% in both the creatine and positive control groups (Figure 5H). This protective effect may be explained by the fact that NooQ5 directly supplies the necessary raw metabolic materials to sustain cell viability under stress. In contrast, while a protective antioxidant effect has been demonstrated when C2C12 cell cultures are pre-treated with creatine prior to the onset of H_2_O_2_ -induced damage [48], this cytoprotection is lost when creatine is administered concurrently or following the oxidative challenge [49]. Under post-stress conditions, creatine transport becomes counterproductive, as injured cells are forced to expend their own diminishing energy reserves to actively internalize the compound, thereby accelerating metabolic exhaustion. On the other hand, in vitro exogenous NAC experiments using H9c2 and primary culture of hippocampal neurons demonstrated a significant decrease in cell viability via MTT assays when cells were exposed to H_2_O_2_ in the presence of NAC compared to non-injury controls [50,51]. This underscores that despite its well-known antioxidant potential, the therapeutic effect of NAC may not fully counteract severe oxidative injury in culture. Nevertheless, its impact on glutathione dynamics remains evident, as NAC serves as a direct precursor in this pathway due to its properties as a thiol-containing antioxidant [50]. As illustrated in Figure 5I, the GSH/GSSG ratio in both the creatine and positive control (NAC) groups was significantly higher than that observed in the NooQ5 group. Alternatively, the lower GSH/GSSG ratio observed in the NooQ5 group suggests that these cells may be utilizing parallel pathways. Biochemically, glucose-6-phosphate dehydrogenase (G6PD) supports cell growth by supplying the ribose and NADPH necessary for nucleotide precursor synthesis [52]. However, supplying nucleotides directly bypasses this enzymatic step. This shortcut is enough to keep cells alive and help them grow, particularly when in a metabolic imbalance. Evidence from rodent models shows that nucleotide supplementation offers strong antioxidant and anti-aging benefits. It is reported that these compounds help maintain cellular balance and protect DNA from damage by directly boosting mitochondrial health and lowering overall oxidative stress [11]. This mitochondrial preservation offers a mechanistic explanation for the reduced LDH leakage and the higher cell viability observed in the NooQ5 group (Figure 5H,J). On the other hand, NAC has been reported to directly inhibit LDH activity in vitro [53].

In the BaCl_2_-induced injury model, NooQ5 promoted a progressive recovery characterized by a higher fusion index and a significant increase in myofiber relative area compared to both the creatine and positive control (IGF-1) groups (Figure 6B,C). These findings indicate that exogenous nucleotide supplementation actively accelerates the differentiation and fusion of myoblasts. This beneficial outcome may be driven by the capacity of NooQ5 to exert a direct amino-acid-sparing effect [7,54]. By providing ready-to-use nucleotides, the cells bypass de novo synthesis, thereby preserving the intracellular pool of amino acids exclusively for protein synthesis and myotube assembly during regeneration.

When comparing the treatment effects, both NooQ5 and creatine induced a progressive increase in differentiated myotube size over time relative to the untreated BaCl_2_-only and the positive control groups (Figure 6C–O). Creatine monohydrate is widely established as a safe and effective ergogenic aid for promoting muscle hypertrophy [55,56]. NooQ5 exhibited a comparable hypertrophic efficiency, as shown by the increased myotube area, and not a hyperplasia effect, as corroborated by the cell viability assay (Figure 1E and Figure 2A,B). NooQ5 exhibited a comparable hypertrophic efficiency, as evidenced by the increased myotube area, rather than a hyperplastic effect, corroborated by the stable cell viability assay results (Figure 1E and Figure 2A,B).

Regarding the transcriptional profile of myogenic regulatory factors, *MyoD* expression was significantly downregulated across almost all damaged groups, whereas the positive control (IGF-1) sustained constant expression at basal levels identical to the undamaged differentiation control (Figure 6P). *MyoD* is known as an early-stage transcription factor responsible for myoblast proliferation [57,58,59]. However, cells in the injury model were kept in differentiation conditions. Therefore, the low *MyoD* transcript levels observed in the NooQ5 and creatine groups at this point suggest that these treatments successfully accelerated the myogenic timeline, driving the structural maturation and fusion stages. On the other hand, IGF-I amplifies downstream hypertrophic cascades, such as the MAPK and Akt/PKB pathways, without requiring sustained *MyoD* upregulation [37]. This mechanism would explain why *MyoD* expression remained at constant basal levels in this specific group.

The transcriptional profile of *desmin* exhibited a distinct biphasic pattern, characterized by an initial increase at 24 h, a transient downregulation at 48 h, and a subsequent upregulation by 96 h (Figure 6Q). In the context of BaCl_2_-induced injury, this kinetic behavior reflects a profound cellular remodeling and adaptation process. The initial levels at 24 h likely represent residual transcripts from the pre-existing myotubes before the chemical insult. At 48 h, cells adapt to acute damage within the supplemented GM. After this, the media is changed to supplemented DM, in which a transitional phase occurs where these damaged structural remnants are degraded, and the monocultures temporarily prioritize survival and metabolic realignment over structural assembly, resulting in the observed downregulation. After 96 h, myotubes resurface due to the myogenic reconstruction observed in the immunofluorescence results.

The progressive increase in extracellular LDH activity observed across all experimental groups over time (Figure 6S) could be attributed to physiological cell turnover and density-dependent stress inherent to prolonged in vitro cultivation [60,61]. Beyond this baseline accumulation, an analysis of CK kinetics revealed distinct metabolic adaptations between treatments. The higher enzyme concentration in the creatine group highlights how exogenous creatine saturation forces myoblasts to overexpress CK (Figure 6R). Conversely, the significantly lower CK levels found in the NooQ5 and IGF-1 groups reflect a more balanced energetic homeostasis that bypasses the need for phosphocreatine-system hyperactivation.

## 5. Conclusions

While further in vivo animal studies are warranted to fully validate its translational efficacy and functional outcomes during muscle contraction, the current literature confirms that dietary nucleotides are non-cytotoxic and already safely utilized to modulate immune responses without adverse effects. Unlike creatine, which demands substrate-induced enzymatic hyperactivation, NooQ5 optimizes cellular recovery via nucleotide salvage pathways without disrupting intracellular homeostasis. Consequently, these findings position NooQ5 as a potent, safe, and innovative alternative for driving structural muscle remodeling.

## Figures and Tables

**Figure 1:**
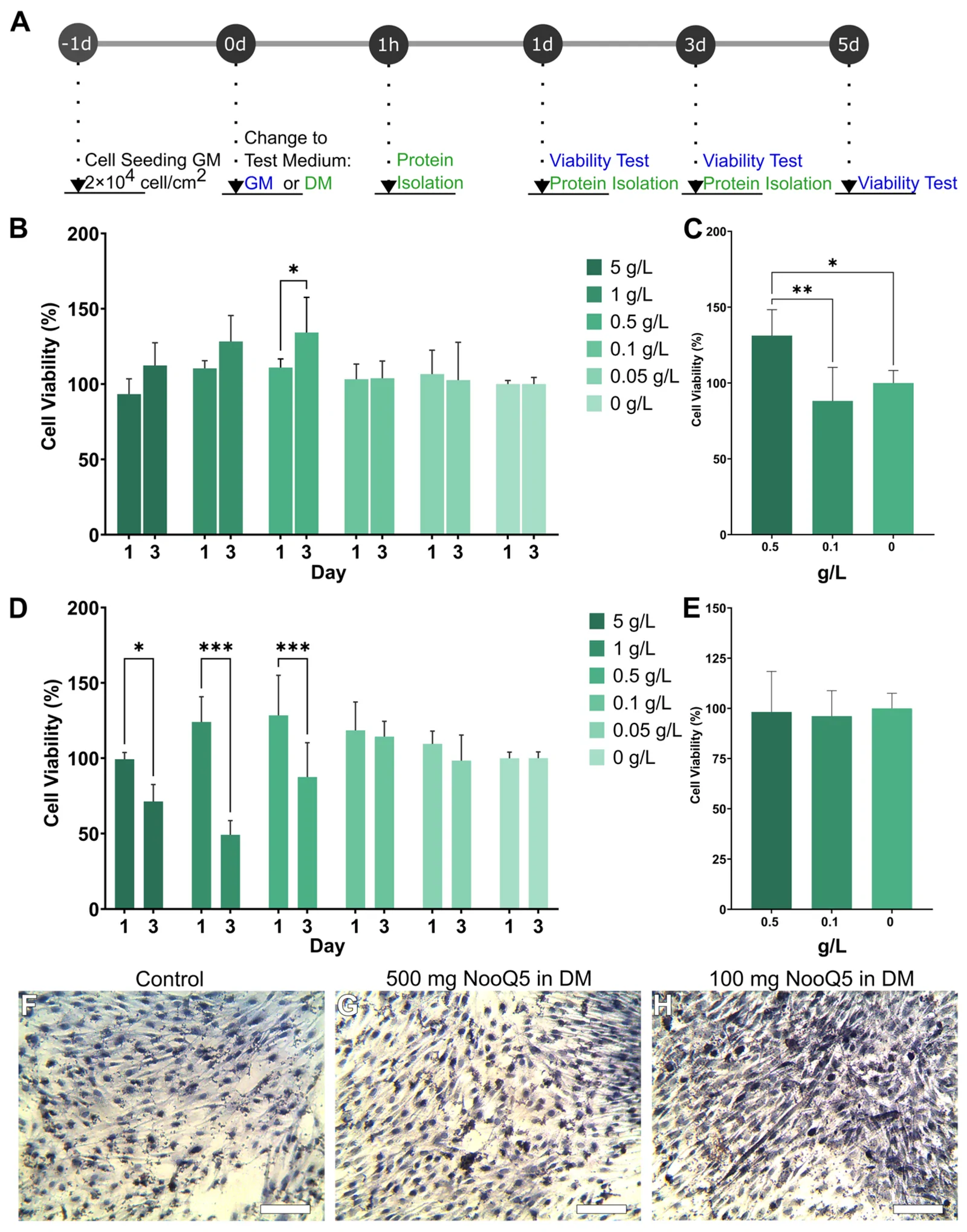
Effects of NooQ5 supplementation on C2C12 cell culture viability, morphology, and differentiation. (**A**) Integrated experimental timeline outlining the short − and long − term screening procedures and analysis points presented across Figure 1, Figure 2 and Figure 3. (**B**) Cell viability evaluated by the MTT assay in C2C12 cells cultured in GM supplemented with various concentrations (0 to 5 g/L) at day 1 and day 3. (**C**) Cell viability in GM at day 5, comparing selected concentrations (0.1 g/L and 0.5 g/L) against the control (0 g/L). (**D**) Cell viability evaluated by MTT assay in C2C12 cells cultured under SM supplemented with various concentrations (0 to 5 g/L) at day 1 and day 3. (**E**) Cell viability in DM at day 5, comparing 0.5 g/L and 0.1 g/L against the control. (**F**–**H**) Immunocytochemistry analysis of C2C12 cells at day 5. (**F**) Control group in DM without supplementation. (**G**) DM supplemented with 0.5 g/L of NooQ5. (**H**) DM supplemented with 0.1 g/L of NooQ5 for 5 days. Myotubes are stained with myosin heavy chains (DAB), and nuclei are counterstained with hematoxylin. Scale bar = 50 µm. Data are presented as mean ± SD. Statistical significance was determined by Sidak’s (for (**B**,**D**)), Tukey’s (for (**C**)), and Dunn’s (for (**E**)) multiple comparisons tests (* *p* ≤ 0.05, ** *p* ≤ 0.01, *** *p* ≤ 0.001).

**Figure 2:**
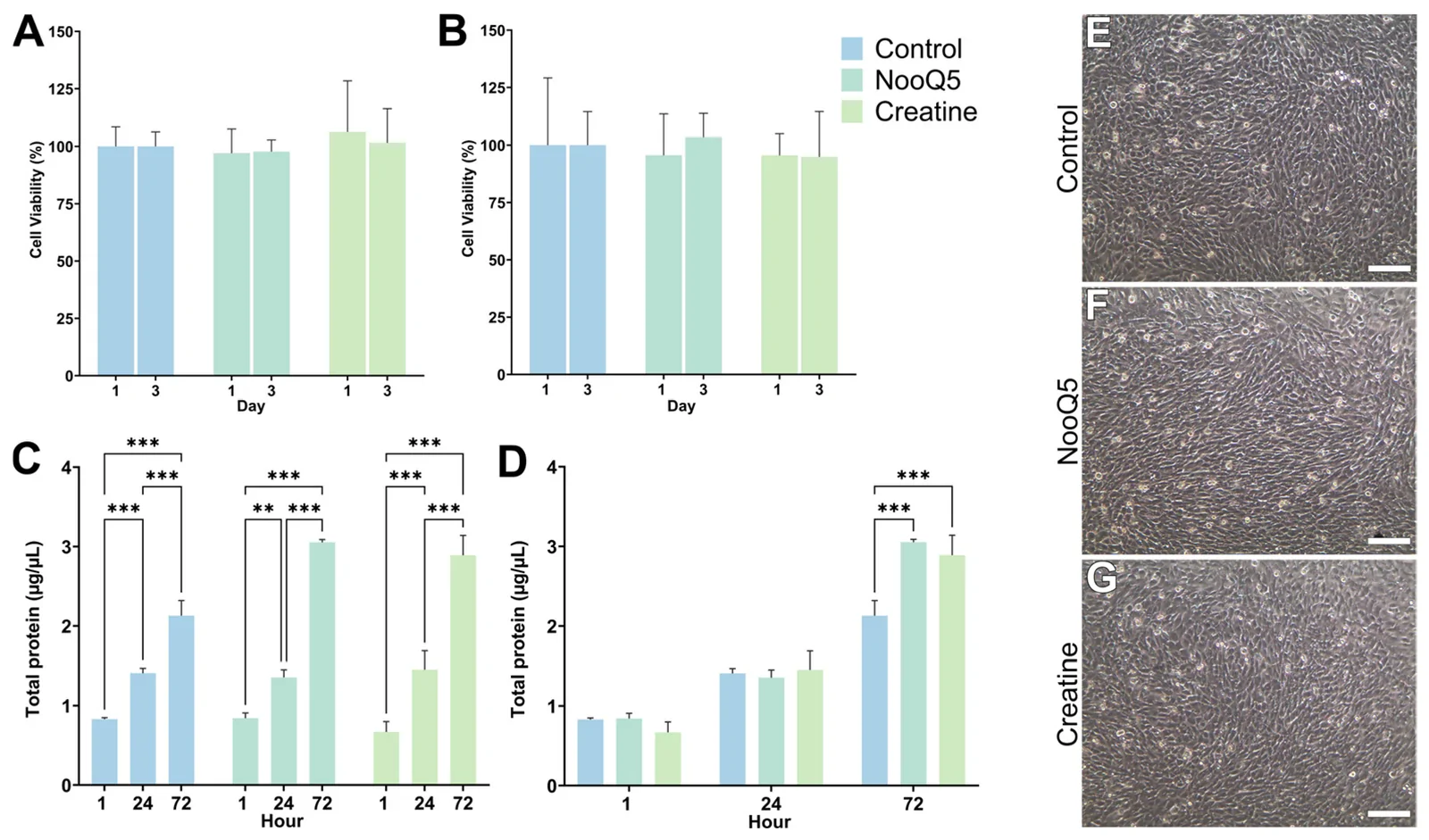
Effects of NooQ5 and creatine supplementation on C2C12 cell viability and morphology. Cell viability evaluated by MTT assay of C2C12 cells in (**A**) GM and (**B**) DM treated with NooQ5 (0.1 g/L) or creatine (0.6 g/L) at days 1 and 3. (**C**,**D**) Total protein quantification in DM conditions using the Bradford assay. (**E**–**G**) Representative phase-contrast microscopy images of C2C12 cultures cultured in GM for 3 days showing cell morphology and confluency under (**E**) control, (**F**) NooQ5, and (**G**) creatine conditions, demonstrating no observable morphological difference. Scale bar = 100 µm. Data are presented as mean ± SD. Statistical significance was determined by Sidak’s (for (**A**,**B**)) and Tukey’s (for (**C**,**D**)) multiple comparisons tests (** *p* ≤ 0.01, *** *p* ≤ 0.001).

**Figure 3:**
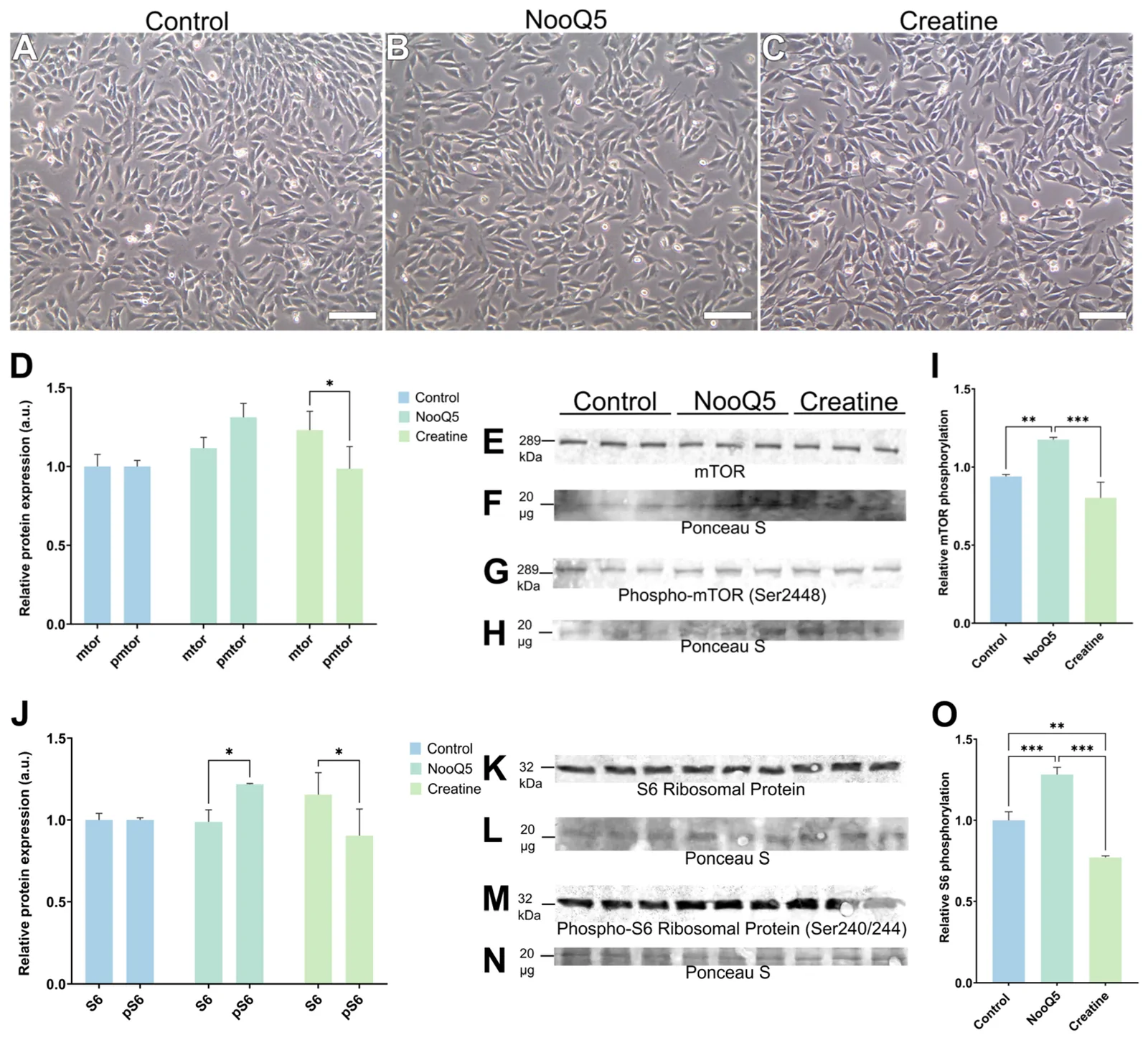
Effects of NooQ5 and creatine supplementation after 1 h of treatment. (**A**–**C**) Representative phase-contrast microscopy images of C2C12 cultures 1 h post-treatment under (**A**) control, (**B**) NooQ5, and (**C**) creatine conditions. (**D**) Relative protein expression of total mTOR and phospho-mTOR (p-mTOR). (**E**,**G**) Representative immunoblot bands for mTOR and p-mTOR (**F**,**H**) with their respective Ponceau S staining as loading controls. (**I**) Relative mTOR phosphorylation ratio (p-mTOR/mTOR). (**J**) Relative protein expression of total S6 and phospho-S6 (p-S6). (**K**,**M**) Representative immunoblot bands for S6 and p-S6, (**L**,**N**) with their respective Ponceau S staining. (**O**) Relative S6 phosphorylation ratio (p-S6/S6). Scale bar = 100 µm. Data are presented as mean ± SD. Statistical significance was determined by Sidak’s (for (**D**,**J**)) and Tukey’s (for (**I**,**O**)) multiple comparisons tests (* *p* ≤ 0.05, ** *p* ≤ 0.01, *** *p* ≤ 0.001).

**Figure 4:**
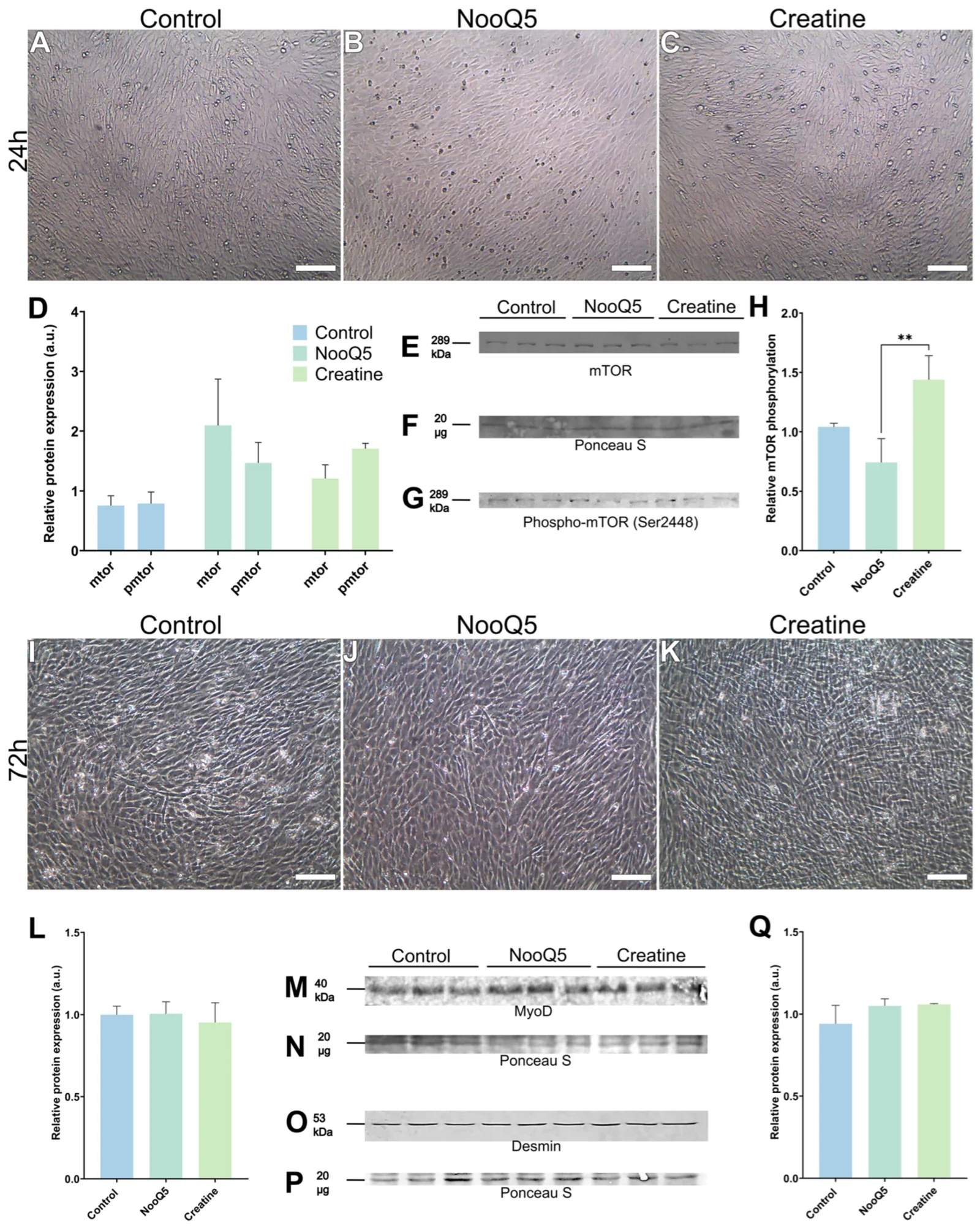
Long-term effects of NooQ5 and creatine supplementation on C2C12 morphology and myogenic regulatory factors. (**A**–**C**) Representative phase-contrast microscopy images of C2C12 cultures after 24 h of differentiation under (**A**) control, (**B**) NooQ5, and (**C**) creatine conditions. (**D**) Relative protein expression of total mTOR and phospho-mTOR (p-mTOR). (**E**,**G**) Representative immunoblot bands for mTOR and p-mTOR, with (**F**) their respective Ponceau S staining as loading controls. (**H**) Relative mTOR phosphorylation ratio (p-mTOR/mTOR). (**I**–**K**) Representative phase-contrast microscopy images after 72 h of differentiation under (**I**) control, (**J**) NooQ5, and (**K**) creatine conditions. (**L**) Relative protein abundance of MyoD at 24 h. (**M**) Representative immunoblots for MyoD at 24 h with (**N**) their respective Ponceau S staining as the loading control. (**Q**) Relative protein abundance of desmin at 72 h. (**O**) Representative immunoblots for desmin at 72 h with (**P**) their respective Ponceau S staining. Scale bar = 100 µm. Data are presented as mean ± SD. Statistical significance was determined by Tukey’s multiple comparisons test (** *p* ≤ 0.01).

**Figure 5:**
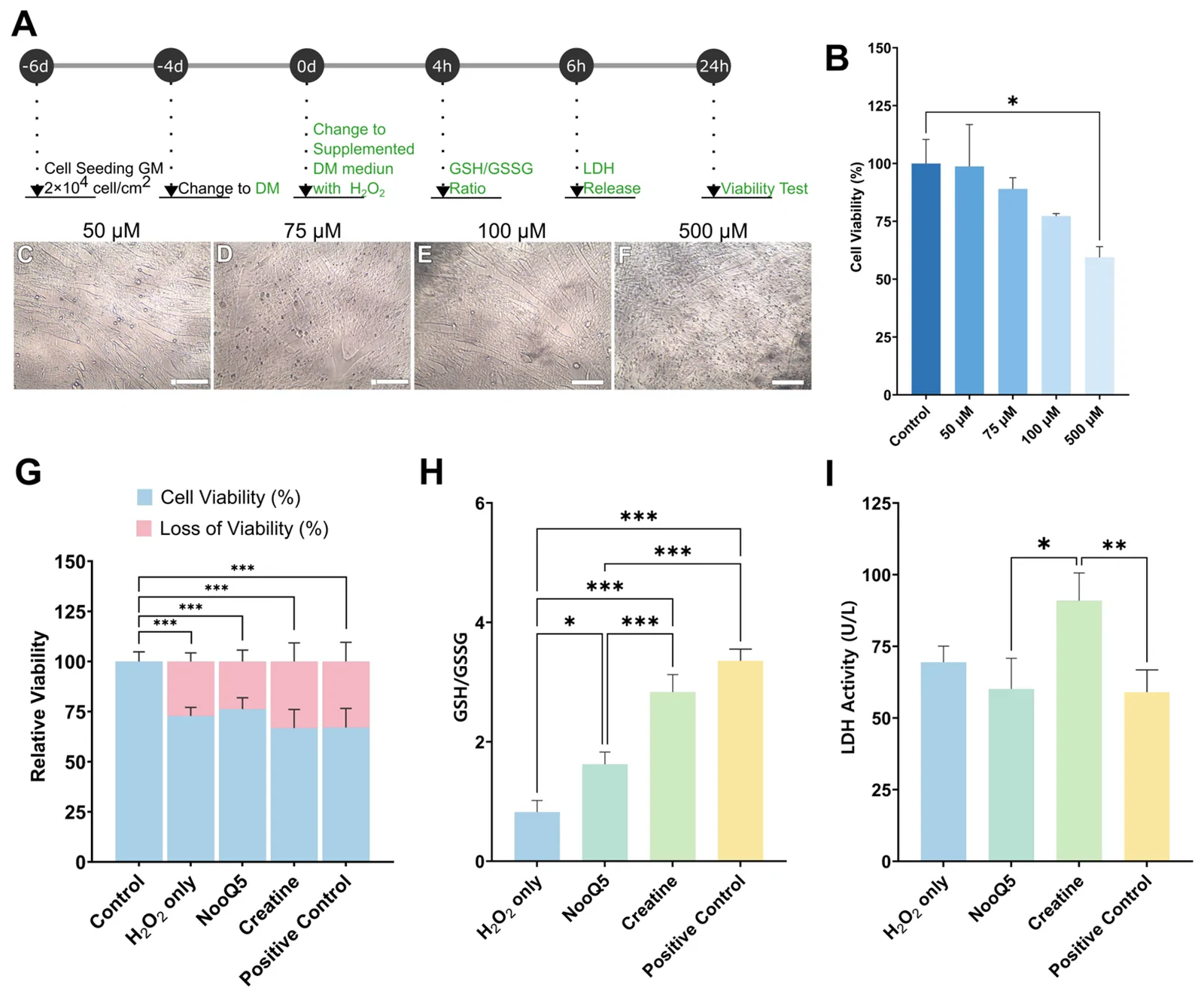
Protective effects of NooQ5 and creatine supplementation against H_2_O_2_-induced oxidative stress in differentiated C2C12 myotubes. (**A**) Timeline of the experimental design for the oxidative stress model. (**B**) Dose–response cell viability curve evaluated by MTT assay to establish the H_2_O_2_ concentration for the oxidative stress model. (**C**–**F**) Representative phase-contrast microscopy images of differentiated C2C12 myotubes exposed to various concentrations of H_2_O_2_: (**C**) 50 µM, (**D**) 75 µM, (**E**) 100 µM, and (**F**) 500 µM. (**G**) Relative cell viability and corresponding loss of viability (%) after H_2_O_2_ injury in cells treated with NooQ5, creatine, or NAC (positive control), compared to the untreated control group. (**H**) Intracellular GSH/GSSG ratio determined 4 h post-injury for H_2_O_2_-only, NooQ5, creatine, and positive control (NAC) groups. (**I**) LDH enzyme activity (U/L) released into the culture medium 6 h post-injury. Scale bar = 100 µm. Data are presented as mean ± SD. Statistical significance was determined by Tukey’s (for (**B**,**H**,**I**)) and Sidak’s (for (**G**)) multiple comparisons tests (* *p* ≤ 0.05, ** *p* ≤ 0.01, *** *p* ≤ 0.001).

**Figure 6:**
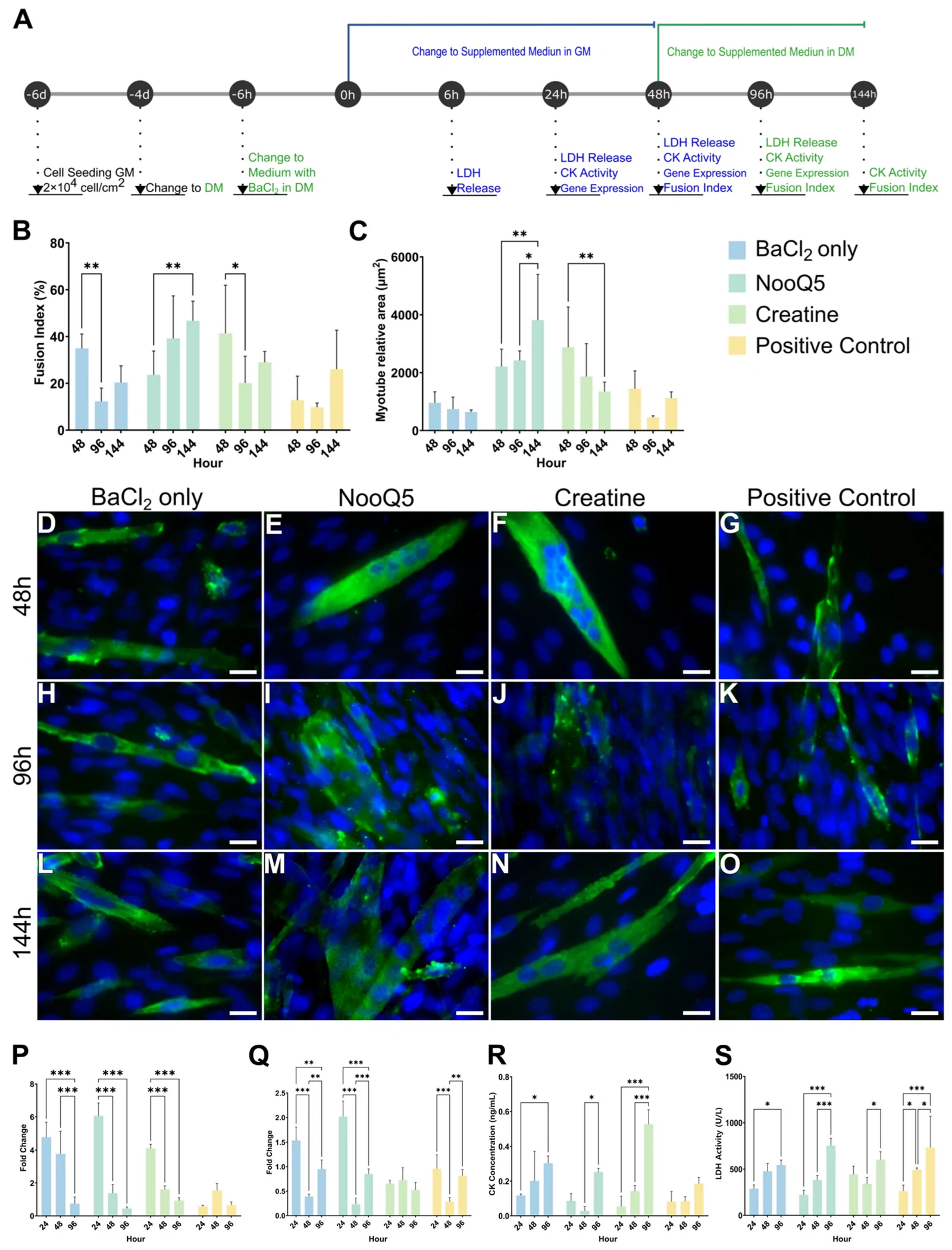
Effects of NooQ5 and creatine supplementation on myotube regeneration and recovery following BaCl_2_-induced injury. (**A**) Timeline of the experimental design for the muscle injury and recovery model, showing the 6 h BaCl_2_ exposure followed by recovery in supplemented GM up to 48 h, and subsequently in supplemented DM up to 144 h. (**B**) Myotube fusion index (%) calculated at 48, 96, and 144 h post-injury. (**C**) Relative myotube size expressed as cross-sectional area (µm^2^) at the same recovery time points. (**D**–**O**) Representative immunofluorescence images of regenerating C2C12 cultures stained for myosin heavy chain (green) and nuclei counterstained with DAPI (blue) at 48 h (**D**–**G**), 96 h (**H**–**K**), and 144 h (**L**–**O**). Panels represent the BaCl_2_-only negative control (**D**,**H**,**L**), NooQ5 treatment (**E**,**I**,**M**), creatine treatment (**F**,**J**,**N**), and IGF-1 as the positive control treatment (**G**,**K**,**O**). (**P**,**Q**) Relative gene expression determined by qPCR for (**P**) *MyoD* and (**Q**) *desmin* at 24, 48, and 96 h post-injury, compared to cells maintained in DM without damage. (**R**) CK concentration (ng/mL) released into the culture medium. (**S**) LDH enzyme activity (U/L) measured in the culture medium at specified time points. Scale bar = 50 µm. Data are presented as mean ± SD. Statistical significance was determined by two-way ANOVA followed by Tukey’s multiple comparisons test (* *p* ≤ 0.05, ** *p* ≤ 0.01, *** *p* ≤ 0.001).

## Data Availability

The data presented in this study are available upon request from the corresponding author, as the complete data cannot be made available due to privacy restrictions.

## References

[1] Berdis A. (2022). Nucleobase-Modified Nucleosides and Nucleotides: Applications in Biochemistry, Synthetic Biology, and Drug Discovery. Front. Chem..

[2] Ding T., Song G., Liu X., Xu M., Li Y. (2021). Nucleotides as Optimal Candidates for Essential Nutrients in Living Organisms: A Review. J. Funct. Foods.

[3] Chandel N.S. (2021). Nucleotide Metabolism. Cold Spring Harb. Perspect. Biol..

[4] Lane A.N., Fan T.W.M. (2015). Regulation of Mammalian Nucleotide Metabolism and Biosynthesis. Nucleic Acids Res..

[5] Dibakoane S.R., Mnisi C.M., Mhlongo G., Wokadala O.C., Mlambo V. (2025). Nucleotides as Conditionally Essential Nutrients for Optimal Health, Performance, and Product Quality in Laying Hens: A Concise Review. Discov. Agric..

[6] Tian L., Xu M. (2026). Exogenous Nucleotides as Functional Food Supplements: A Bibliometric Analysis of Global Research Trends (2000–2025). Foods.

[7] Koziara Z., Kościelak A., Cifani C., Bartoszek A. (2026). Dietary Supplements Containing Nucleic Acids, Nucleotides and Nucleosides; Availability, Composition, Suggested Health Effects. Food Biosci..

[8] Riera J., Pons V., Martinez-Puig D., Chetrit C., Tur J.A., Pons A., Drobnic F. (2013). Dietary Nucleotide Improves Markers of Immune Response to Strenuous Exercise under a Cold Environment. J. Int. Soc. Sports Nutr..

[9] Ostojic S.M., Obrenovic M. (2012). Sublingual Nucleotides and Immune Response to Exercise. J. Int. Soc. Sports Nutr..

[10] Sterczala A.J., Dupont W.H., Comstock B.A., Flanagan S.D., Szivak T.K., Hooper D.R., Kupchak B.R., Lee E.C., Volek J.S., Maresh C.M. (2016). Physiological Effects of Nucleotide Supplementation on Resistance Exercise Stress in Men and Women. J. Strength Cond. Res..

[11] Gene-Morales J., Juesas A., Saez-Berlanga A., Martin E.G., Garrigues-Pelufo L., Sandoval-Camargo B.S., Martin-Rivera F., Chulvi-Medrano I., Jiménez-Martínez P., Alix-Fages C. (2025). Dietary Nucleotides Enhance Neurogenesis, Cognitive Capacity, Muscle Function, and Body Composition in Older Adults: A Randomized, Triple-Blind, Controlled Clinical Trial. Nutrients.

[12] Xu M., Liang R., Li Y., Wang J. (2017). Anti-Fatigue Effects of Dietary Nucleotides in Mice. Food Nutr. Res..

[13] Valini G.A.C., Duarte M.S., Calderano A.A., Teixeira L.M., Rodrigues G.A., Fernandes K.M., Veroneze R., Serão N.V.L., Mantovani H.C., Rocha G.C. (2021). Dietary Nucleotide Supplementation as an Alternative to In-Feed Antibiotics in Weaned Piglets. Animal.

[14] Waititu S.M., Yin F., Patterson R., Yitbarek A., Rodriguez-Lecompte J.C., Nyachoti C.M. (2017). Dietary Supplementation with a Nucleotide-Rich Yeast Extract Modulates Gut Immune Response and Microflora in Weaned Pigs in Response to a Sanitary Challenge. Animal.

[15] Abbaslou Y., Zahmatkesh D., Mahjoubi E., Yazdi M.H., Beiranvand H., Gorjidooz M. (2021). Nucleotide Supplementation to Whole Milk Has Beneficial Effects on Post-Weaning Holstein Calf Performance. Animals.

[16] Bonagurio L.P., Murakami A.E., Moreira C.A., Comar J.F., Pozza P.C. (2022). Dietary Supplementation with Inosine-5′-Monophosphate Improves the Functional, Energetic, and Antioxidant Status of Liver and Muscle Growth in Pigs. Sci. Rep..

[17] de Souza C., Eyng C., Viott A.M., de Avila A.S., Pacheco W.J., Junior N.R., Kohler T.L., Tenorio K.I., Cirilo E.H., Nunes R.V. (2021). Effect of Dietary Guanidinoacetic Acid or Nucleotides Supplementation on Growth Performances, Carcass Traits, Meat Quality and Occurrence of Myopathies in Broilers. Livest. Sci..

[18] Wang Y., Wang J., Liu L., Xu H., Liang H., Wang Z., Gu J. (2024). Effects of Dietary Yeast Nucleotides on the Growth Performance and Muscle Quality of Juvenile Largemouth Bass (*Micropterus salmoides*). Aquac. Rep..

[19] Hassanein I.I., Metwally A.E., Elbaky H.E.M.A. (2023). Effects of dietary nucleotides supplementation on the perfor-mance of broiler chicks. Slov. Vet. Res..

[20] Wu X., Liu R., Zhu N., Wang X., Wei C., An X., Xu M., Li Y. (2025). Exogenous Nucleotides Supplementation Attenuates Age-Related Sarcopenia. J. Cachexia Sarcopenia Muscle.

[21] Candow D.G., Moriarty T. (2024). Effects of Creatine Monohydrate Supplementation on Muscle, Bone and Brain- Hope or Hype for Older Adults?. Curr. Osteoporos. Rep..

[22] Stout J.R., Kreider R.B., Candow D.G., Forbes S.C., Rawson E.S., Antonio B., Antonio J. (2025). The Birth of Modern Sports Nutrition: Tracing the Path from Muscle Biopsies to Creatine Supplementation—A Narrative Review. J. Int. Soc. Sports Nutr..

[23] Wu S.H., Chen K.L., Hsu C., Chen H.C., Chen J.Y., Yu S.Y., Shiu Y.J. (2022). Creatine Supplementation for Muscle Growth: A Scoping Review of Randomized Clinical Trials from 2012 to 2021. Nutrients.

[24] Pierre N., Barbé C., Gilson H., Deldicque L., Raymackers J.M., Francaux M. (2014). Activation of ER Stress by Hydrogen Peroxide in C2C12 Myotubes. Biochem. Biophys. Res. Commun..

[25] Siu P.M., Wang Y., Alway S.E. (2009). Apoptotic Signaling Induced by H_2_O_2_-Mediated Oxidative Stress in Differentiated C2C12 Myotubes. Life Sci..

[26] El Assar M., Álvarez-Bustos A., Sosa P., Angulo J., Rodríguez-Mañas L. (2022). Effect of Physical Activity/Exercise on Oxidative Stress and Inflammation in Muscle and Vascular Aging. Int. J. Mol. Sci..

[27] Raizada S., Jyotika, Kushwaha S., Shrivastava R. (2026). Revisiting Barium Chloride-Induced Injury as a Preclinical Model for Skeletal Muscle Regeneration. Tissue Cell.

[28] Tarum J., Degens H., Turner M.D., Stewart C., Sale C., Santos L. (2023). Modelling Skeletal Muscle Ageing and Repair In Vitro. J. Tissue Eng. Regen. Med..

[29] Kase N., Kitagawa Y., Ikenaka A., Niwa A., Saito M.K. (2023). A Concise in Vitro Model for Evaluating Interactions between Macrophage and Skeletal Muscle Cells during Muscle Regeneration. Front. Cell Dev. Biol..

[30] Fleming J.W., Capel A.J., Rimington R.P., Player D.J., Stolzing A., Lewis M.P. (2019). Functional Regeneration of Tissue Engineered Skeletal Muscle in Vitro is Dependent on the Inclusion of Basement Membrane Proteins. Cytoskeleton.

[31] Mäntyselkä S., Kolari K., Baumert P., Yla-Outinen L., Kuikka L., Lahtonen S., Permi P., Wackerhage H., Kalenius E., Kivela R. (2024). Serine Synthesis Pathway Enzyme PHGDH Is Critical for Muscle Cell Biomass, Anabolic Metabolism, and MTORC1 Signaling. Am. J. Physiol. Endocrinol. Metab..

[32] Mäntyselkä S., Ahvenlammi M., Vartiainen J., Halonen E.J., Kolari K., Wackerhage H., Permi P., Varjosalo M., Kelahaara M.M., Ahtiainen J.P. (2025). Glycolytic Metabolism and Biomass Production from Glucose in Human Skeletal Muscle Growth. Am. J. Physiol. Endocrinol. Metab..

[33] Lunt S.Y., Vander Heiden M.G. (2011). Aerobic Glycolysis: Meeting the Metabolic Requirements of Cell Proliferation. Annu. Rev. Cell Dev. Biol..

[34] Tedeschi P.M., Markert E.K., Gounder M., Lin H., Dvorzhinski D., Dolfi S.C., Chan L.L.Y., Qiu J., DiPaola R.S., Hirshfield K.M. (2013). Contribution of Serine, Folate and Glycine Metabolism to the ATP, NADPH and Purine Requirements of Cancer Cells. Cell Death Dis..

[35] Vidal-Cruchez O., Ben-Sahra I. (2026). The Oncogenic Control of Nucleotide Synthesis. Oncogenesis.

[36] Diehl F.F., Miettinen T.P., Elbashir R., Nabel C.S., Darnell A.M., Do B.T., Manalis S.R., Lewis C.A., Vander Heiden M.G. (2022). Nucleotide Imbalance Decouples Cell Growth from Cell Proliferation. Nat. Cell Biol..

[37] Deldicque L., Theisen D., Bertrand L., Hespe P., Hue L., Francaux M. (2007). Creatine Enhances Differentiation of Myogenic C2C12 Cells by Activating Both P38 and Akt/PKB Pathways. Am. J. Physiol. Cell Physiol..

[38] Mao X., Kelty T.J., Kerr N.R., Childs T.E., Roberts M.D., Booth F.W. (2021). Creatine Supplementation Upregulates MTORC1 Signaling and Markers of Synaptic Plasticity in the Dentate Gyrus While Ameliorating LPS-Induced Cognitive Impairment in Female Rats. Nutrients.

[39] Pham B.Q., Yi S.A., Ordureau A., An H. (2025). MTORC1 Regulates the Pyrimidine Salvage Pathway by Controlling UCK2 Turnover via the CTLH-WDR26 E3 Ligase. Cell Rep..

[40] Sun M., Jiao H., Wang X., Li H., Zhou Y., Zhao J., Lin H. (2022). The Regulating Pathway of Creatine on Muscular Protein Metabolism Depends on the Energy State. Am. J. Physiol. Cell Physiol..

[41] Gao L.M., Zhou T.T., Chen Z.P., Wassie T., Li B., Wu X., Yin Y.L. (2021). Maternal Yeast-Based Nucleotide Supplementation Decreased Stillbirth by Regulating Nutrient Metabolism. J. Sci. Food Agric..

[42] Wu X., Zhu N., He L., Xu M., Li Y. (2024). 5′-Cytimidine Monophosphate Ameliorates H_2_O_2_-Induced Muscular Atrophy in C2C12 Myotubes by Activating IRS-1/Akt/S6K Pathway. Antioxidants.

[43] Hakibilen C., Delort F., Daher M.T., Joanne P., Cabet E., Cardoso O., Bourgois-Rocha F., Tian C., Rivas E., Madruga M. (2022). Desmin Modulates Muscle Cell Adhesion and Migration. Front. Cell Dev. Biol..

[44] Weichhart T. (2018). MTOR as Regulator of Lifespan, Aging, and Cellular Senescence: A Mini-Review. Gerontology.

[45] Kasar G.N., Rasal P.B., Upaganlawarb A.B., Mahajan M.S. (2026). MTOR Signaling in Aging and Longevity: Therapeutic Opportunities. Aging Pathobiol. Ther..

[46] Mannick J.B., Lamming D.W. (2023). Targeting the Biology of Aging with MTOR Inhibitors. Nat. Aging.

[47] Cadena Sandoval M., Heberle A.M., Rehbein U., Barile C., Ramos Pittol J.M., Thedieck K. (2021). MTORC1 Crosstalk with Stress Granules in Aging and Age-Related Diseases. Front. Aging.

[48] Sestili P., Barbieri E., Martinelli C., Battistelli M., Guescini M., Vallorani L., Casadei L., D’Emilio A., Falcieri E., Piccoli G. (2009). Creatine Supplementation Prevents the Inhibition of Myogenic Differentiation in Oxidatively Injured C2C12 Murine Myoblasts. Mol. Nutr. Food Res..

[49] Mahajan A.S., Arikatla V.S., Thyagarajan A., Zhelay T., Sahu R.P., Kemp M.G., Spandau D.F., Travers J.B. (2021). Creatine and Nicotinamide Prevent Oxidant-Induced Senescence in Human Fibroblasts. Nutrients.

[50] Liu X., Wang L., Cai J., Liu K., Liu M., Wang H., Zhang H. (2019). N-Acetylcysteine Alleviates H_2_O_2_-Induced Damage via Regulating the Redox Status of Intracellular Antioxidants in H9c2 Cells. Int. J. Mol. Med..

[51] Wu W., Liu B.H., Xie C.L., Xia X.D., Zhang Y.M. (2018). Neuroprotectiee Effects of N-Acetyl Cysteine on Primary Hippocampus Neurons against Hydrogen Peroxide-Induced Injury Are Mediated Eia Inhibition of Mitogen-Actieated Protein Kinases Signal Transduction and Antioxidatiee Action. Mol. Med. Rep..

[52] Zhang Y., Xu Y., Lu W., Ghergurovich J.M., Guo L., Blair I.A., Rabinowitz J.D., Yang X. (2021). Upregulation of Antioxidant Capacity and Nucleotide Precursor Availability Suffices for Oncogenic Transformation. Cell Metab..

[53] Sun Y., Pu L.Y., Lu L., Wang X.H., Zhang F., Rao J.H. (2014). N-Acetylcysteine Attenuates Reactive-Oxygen-Species-Mediated Endoplasmic Reticulum Stress during Liver Ischemia-Reperfusion Injury. World J. Gastroenterol..

[54] Lopez-Navarro A.T., Ortega M.A., Peragon J., Bueno J.D., Gil A., Sanchez-Pozo A. (1996). Deprivation of Dietary Nucleotides Decreases Protein Synthesis in the Liver and Small Intestine in Rats. Gastroenterology.

[55] Casciola R., Leoni L., Cuffari B., Pecchini M., Menozzi R., Colecchia A., Ravaioli F. (2023). Creatine Supplementation to Improve Sarcopenia in Chronic Liver Disease: Facts and Perspectives. Nutrients.

[56] Vieira P.d.C., Spineli M.N., Bagri K.M., Mendonça H.C., Cibreiros M.P.C., Leão M.H.C., Mermelstein C., Costa M.L. (2026). Creatine Increases Muscle Fiber Size in Embryonic Chick Muscle Cells and Age-Dependent Swimming Performance in Zebrafish. Sci. Rep..

[57] Hu P., Geles K.G., Paik J.H., DePinho R.A., Tjian R. (2008). Codependent Activators Direct Myoblast-Specific MyoD Transcription. Dev. Cell.

[58] Tsuji R., Hayashi T., Takahashi S., Asakura A., Fujita R. (2025). The Multifaceted Role of MyoD in Adult Skeletal Muscle: Homeostasis, Regeneration, and Diseases. Am. J. Physiol. Cell Physiol..

[59] Battistelli C., Garbo S., Maione R. (2022). MyoD-Induced Trans-Differentiation: A Paradigm for Dissecting the Molecular Mechanisms of Cell Commitment, Differentiation and Reprogramming. Cells.

[60] Castiglione H., Madrange L., Lemonnier T., Deslys J.P., Yates F., Vigneron P.A. (2024). Development and Optimization of a Lactate Dehydrogenase Assay Adapted to 3D Cell Cultures. Organoids.

[61] Legrand C., Bour J.M., Jacob C., Capiaumont J., Martial A., Marc A., Wudtke M., Kretzmer G., Demangel C., Duval D. (1992). Lactate Dehydrogenase (LDH) Activity of the Cultured Eukaryotic Cells as Marker of the Number of Dead Cells in the Medium [Corrected]. J. Biotechnol..

